# Evaluation of fracture resistance and marginal fit of implant-supported interim crowns fabricated by conventional, additive and subtractive methods

**DOI:** 10.1186/s12903-024-04597-9

**Published:** 2024-07-27

**Authors:** Safaa Salah Elsareef, Amir Shokry Azer, Noha Morsy

**Affiliations:** 1 Senior researcher of Fixed prosthodontics, Department of conservative Dentistry, Faculty of Dentistry, Alexandria University, Alexandria, Egypt; 2 Associate professor of Fixed prosthodontics, Department of conservative Dentistry, Faculty of Dentistry, Alexandria University, Alexandria, Egypt; 3 Lecturer of Fixed prosthodontics, Department of conservative Dentistry, Faculty of Dentistry, Alexandria University, Alexandria, Egypt

**Keywords:** 3D printing, Implant-supported interim crowns: fracture resistance, Marginal fit, Direct microscopic evaluation, CAD CAM milling, Laboratory analogues

## Abstract

**Background:**

Interim crowns are utilized for restoring implants during and after the process of osseointegration. However, studies on adaptation and fracture strength of implant-supported interim crowns are rare.

**Aim of the study:**

The aim of this in vitro study is evaluating marginal fit and fracture resistance of conventional, subtractive, and additive methods of fabricating implant-supported interim crowns.

**Materials and methods:**

An implant was placed in an epoxy resin model with a missing first molar. A scan body was attached, and scanned with an intraoral scanner (IOS), the STL file was used to fabricate eighteen master models with standardized implant digital analogue spaces. The digital analogues and their corresponding abutments were attached to the master models and scanned with the IOS, the STL files were used to fabricate eighteen crowns using three different techniques (*n* = 6): conventional (CR); from Autopolymerizing composite resin, subtractive (SM); milled from PMMA resin blanks, and additive (AM); from 3D printed resin material. The crowns were fitted and cemented on their corresponding abutments and subjected to cyclic loading and thermocycling. The marginal fit was evaluated using a stereomicroscope. The crowns were then loaded until fractured in a universal testing machine. The Shapiro–Wilk and the Kolmogorov-Smirnov tests revealed that data of Marginal gap was non-parametric. Kruskal-Wallis test followed by the Dunn test was used (α = 0.05). While data of Fracture resistance test was parametric. ANOVA (F-test) was used followed by the Tukey test (α = 0.05).

**Results:**

For marginal gap, a significant difference was shown between the study groups (*P* = .001) according to Kruskal–Wallis test. Groups SM and AM had significantly lower marginal gap values compared to group CR (*P* = .003). No significant difference was found between groups SM and AM (*P* = .994). For fracture resistance, One-way ANOVA revealed a significant difference in fracture resistance between study groups (*P* < .001). Group SM had significantly higher fracture strength followed by group AM and group CR (*P* = .001).

**Conclusions:**

Group SM and AM showed better marginal adaptation than group CR. Group SM showed superior fracture resistance compared to other groups. All study groups showed acceptable marginal gap and fracture resistance.

## Background

Implant-supported interim restoration has a vital role in restoring function till the definitive prosthesis is delivered [1]. In addition, interim restorations are used during healing period of dental implants and shaping of the soft tissue around the implant and subsequently can affect the final esthetic results [2]. Moreover, the interim restoration restores occlusal relationship and protect the abutments in cases requiring long term healing and progressive loading [3]. The normal forces generated by natural teeth during occlusion range from 200 to 900 N [4]. It was reported that patients with implant-supported restoration have poor proprioception and may experience higher forces [5]. Therefore, implant-supported interim restorations should withstand such forces. Moreover, they should have accurate margins for soft-tissue modeling and avoiding periimplantitis [5, 6].

Material selection and fabrication techniques are essential factors impacting the performance of interim restorations. Chairside direct fabrication of interim crowns is performed conventionally using di-methacrylates. Although conventional fabrication is available and cost-effective, it has its drawbacks. The conventional crowns were reported to have inferior surface texture, high volumetric shrinkage, longer time required in dental chair, and the inclusion of air bubbles during mixing and dispensing process [7, 8].

Interim restorations can be fabricated with computer-aided manufacturing (CAM) technology either with subtractive (SM) or additive manufacturing (AM) [9]. The CAM interim restorations were reported to have greater durability, better fit, and color stability [10]. SM restorations are milled from highly cross-linked blanks and reported to have superior mechanical properties [10, 11]. Despite the advantages of milled interim crowns, milling machines have limitations including material waste, microcracks, and limitations related to milling burs size [12]. On the other hand, additive manufacturing reduces waste, with more availability of machines and materials used, and the ability to fabricate complex structures accurately. However, 3D-printed objects have limited color options and exhibit a staircase effect [13]. Many factors affect the additive manufacturing technology such as slicing, geometry of the device, build platform position and angle, and post-processing procedures [13–20].

Several studies have assessed the fracture resistance and marginal fit of milled and 3D-printed interim restorations and the results were contradictory [4, 7, 20–28]. However, the studies regarding implant-supported interim crowns are scarce and contradictory. Martin-Orteg et al. [4] reported better fracture strength for milled implant-supported interim crowns compared with printed crowns. Another study by Park et al. {6] reported that printed implant-supported interim crowns had better marginal and internal fit than milled and conventional interim crowns. In contrast, a similar study by [29] reported better marginal fit for conventional implant-supported interim crowns than printed and milled crowns. Therefore, this study aimed to compare the marginal fit and fracture resistance of conventionally fabricated, milled, and 3D-printed implant-supported interim crowns. The null hypotheses were that no significant difference would be found between study groups.

## Materials and methods

### Study design and sample size

The study design was in-vitro, parallel controlled where marginal fit and fracture resistance of three parallel groups were examined. It was held at the Conservative Dentistry Department laboratory at the Faculty of Dentistry, Alexandria University, Egypt.

Sample size was estimated using a software program (G* Power Version 3.1.9.4 Department of Biomedical Informatics and Medical Statistics, Medical Research Institute, University of Alexandria, Egypt.) assuming 80% study power and 5% alpha error. Based on comparison of means of a previous study [4], the minimum sample size was calculated to be 5 increased to 6 to make up for laboratory processing errors. The total required sample size = number of groups × number per group = 3 × 6 = 18.

### Preparation of the master model

A stone model of a patient with a missing left lower first molar was used to fabricate the master model for this study. An impression for the stone model was obtained with polyvinylsiloxane impression material was made (Imprint II Monophase VPS Impression Material; 3 M; Saint Paul; USA). The impression was poured with epoxy resin (Epoxy resin HT 111; WEICON GmbH & Co. KG, China) to obtain a master model with a missing left lower first molar. An implant with 4.2 × 10 mm (MPI implant; VitroNex Elite implant, Italy) was placed replacing the missing tooth with the implant shoulder positioned at the gingival level to mimic the bone level placement in a clinical situation.

### Scanning of the implants and printing of the 3D models

**Fig. 1:**
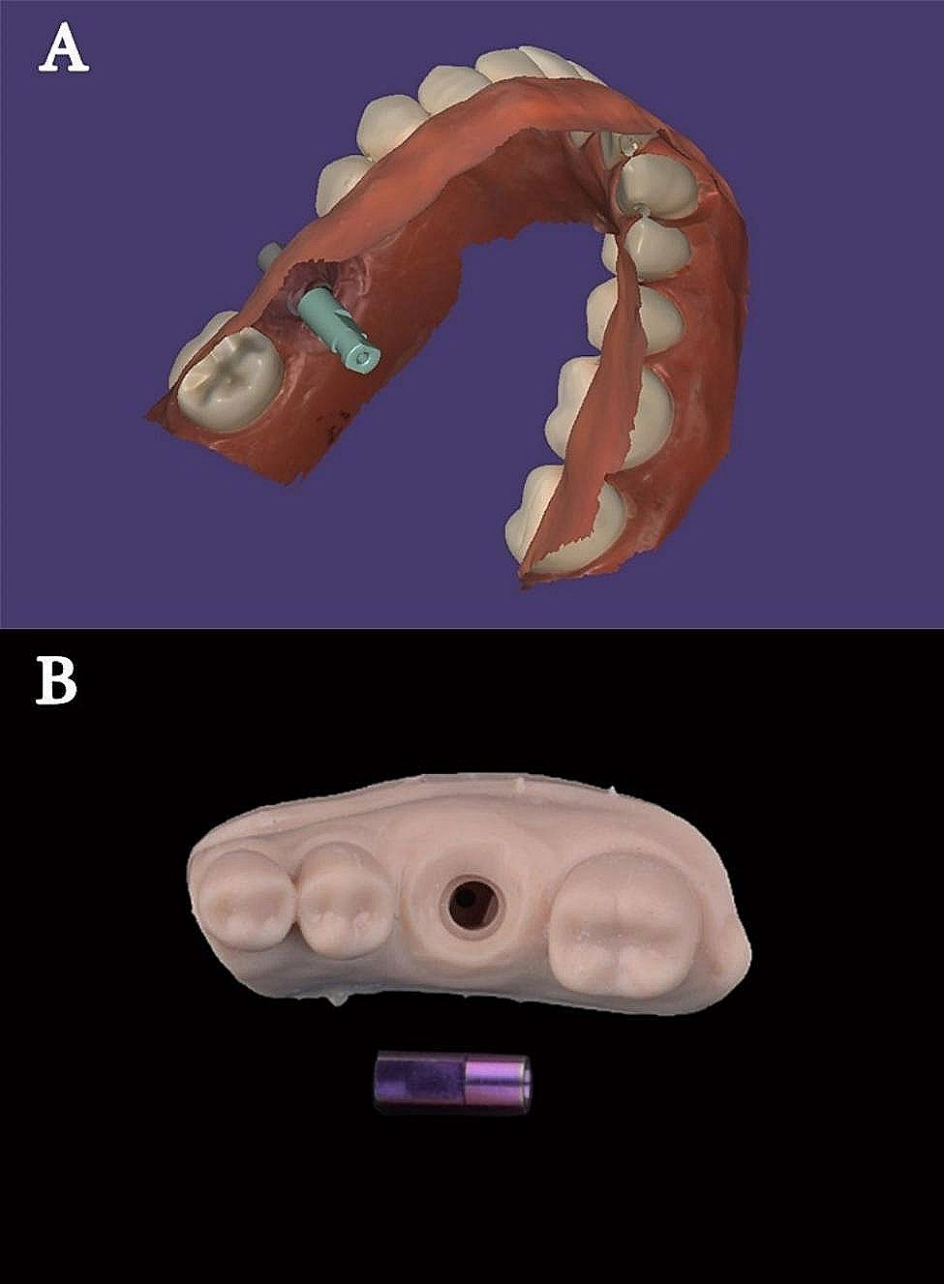
**(A)** Digital implant analogue from CAD software library aligned with 3D model. **(B)** Digital implant laboratory analogue and printed model

A scan body (MPI scan body; VitroNex Elite implant, Italy) was attached to the implant and scanned using an intraoral scanner (IOS) (Medit i700; Medit Corp., Seoul, South Korea) following the scanning protocol recommended by the manufacturer. The occlusal surfaces of the teeth were scanned starting from the last molar of one side to the last molar of the other side, followed by scanning the lingual surfaces then the labial surfaces. The STL file was saved and exported to a CAD software program (Exocad v2.2 Valletta; exocad GmbH, Darmstadt, Germany) to align a digital implant analogue from the software library onto the 3D model (Fig. 1A). The data was exported to a 3-dimensional (3D) printer (NextDent 5100; NextDent B.V., Soesterberg, Netherlands) to fabricate 18 solid master models (Savoy dental model skin; Savoy digital systems, China) with standardized implant digital analogue hollow spaces. Digital laboratory analogues (MPI Lab Analouge; VitroNex Elite implant, Italy) were fitted manually into the corresponding spaces in the 3D printed master models (Fig. 1B). The abutments (MPI Straight Abutment; VitroNex Elite implant, Italy) with a height of 5 mm and a shoulder of 0.5 mm were attached to the analogues and tightened to 30 N/cm according to the recommendations of the manufacturer. The channels for screw access were sealed with flowable composite resin (Nexcomp Flow; META BIOMED, China).

### Scanning of the implant abutments and designing of the interim crowns

Medit i700 IOS was used to scan the abutments, and the resulting STL files were exported to a CAD software program (Exocad v2.2 Valletta; exocad GmbH, Darmstadt, Germany) to design 18 interim crowns. The minimum thickness was adjusted to 1 mm axially and 1.5 mm occlusally. The cement spacer was adjusted to 50 μm 1 mm occlusal to the finish line according the manufacturer recommendations.

### Grouping of specimens

The designs of the interim crowns were assigned into 3 groups (*n* = 6) according to the proceeding manufacturing technique: group CR (conventional composite resin), group SM (subtractive milled polymethylmethacrylate (PMMA)), and group AM (additive 3D-printed resin). The designed crowns were saved as STL files.

### Group CR: fabrication of interim crowns using conventional technique (*n* = 6)

**Fig. 2:**
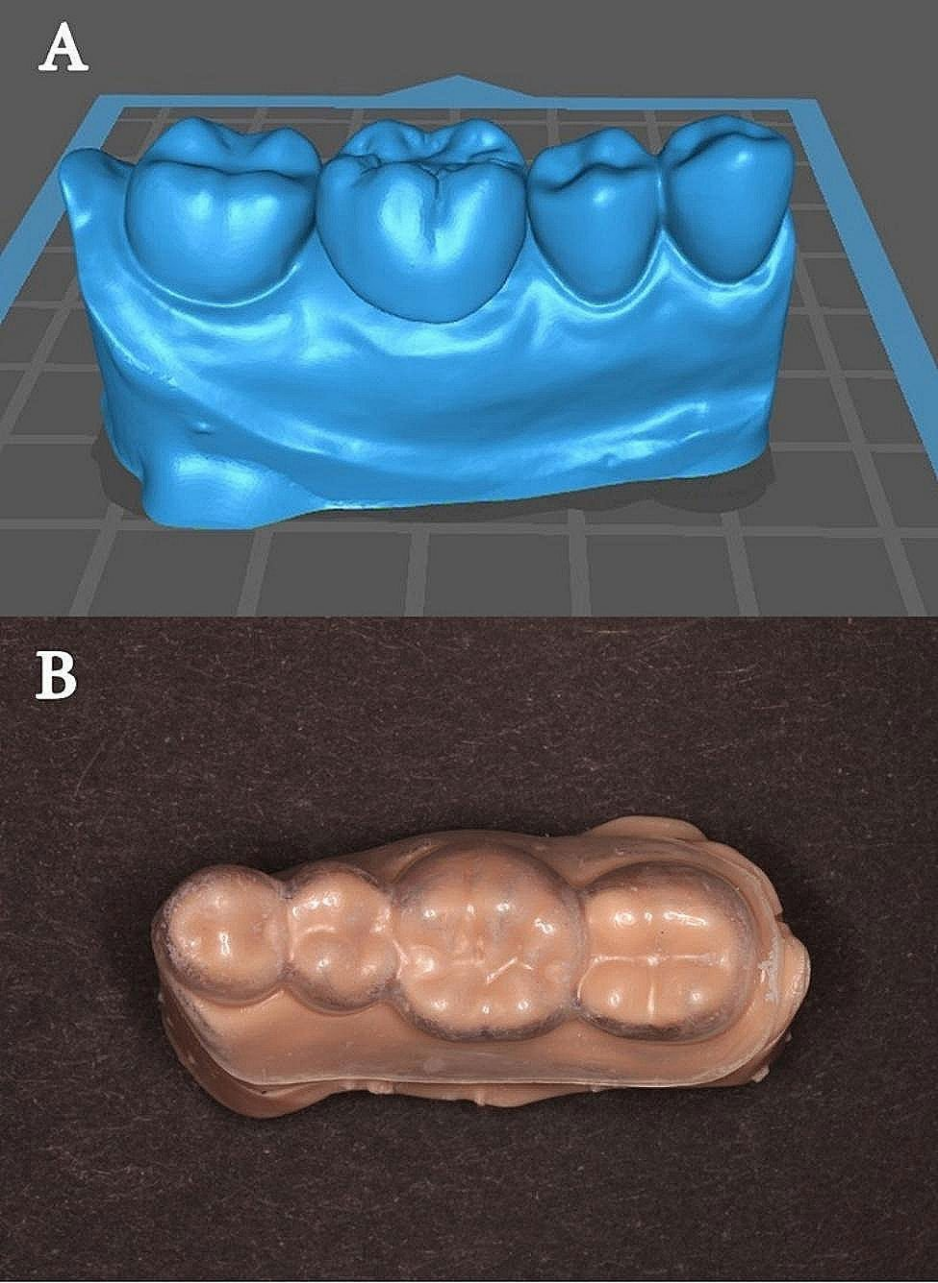
**(A)** Virtual model for template fabrication. **(B)** 3D printed model with vacuum sheet template

For group CR, the designed crowns were digitally merged on their virtual master models to create 6 template models to be used as the external surface forms. The template models were 3D-printed with a printable model resin material (Savoy dental model skin; Savoy digital systems, China) and used to fabricate 6 external surface forms from silicone vacuum sheets (vaccum forming materials; YOU DENT CO., LTD., China) (Fig. 2). The sheets were fitted on their corresponding abutments. Autopolymerizing composite resin (Structure 2; Voco GmbH, Germany) was used to fabricate the conventional interim crowns according to the instructions of manufacturer. The provisional material was automixed (Dispensing gun type 2; Voco GmbH, Germany) with a ratio of 1:1 and dispensed into the templates then seated on the abutments and left to set for 1.5 min. The interim crowns were finished and polished (LD2746; Komet, SC, USA).

### Group SM: fabrication of interim crowns using subtractive milling technique (*n* = 6)

For group SM, the STL files of the designed interim crowns were imported to milling machine (DWX-52D Plus; Roland DG Corp, Japan), and the crowns were milled from PMMA resin blanks (Yamahachi; Aichi co., Japan) as recommended by the manufacturer.

### Group AM: fabrication of interim crowns using additive printing technique (*n* = 6)

For group AM, the STL files of the designs of interim crowns were imported to a 3D-printing software (RapidForm XOR2; 3D Systems Inc., USA) and the crowns were printed from resin material (Savoy C&B resin; Savoy digital system, China) by using a printer with DLP technology (NextDent 5100; NextDent Co., Soesterberg, Netherlands). The specimens were printed with a wavelength of 405 nm, a resolution of 50 μm, and a building speed of 30 mm/h according to the instructions of the manufacturer. The printed specimens were cleaned in ethanol for 5 min, finished, and polished (LD2746; Komet, SC, USA), and post-cured (LC-3DPrint Box; NextDent Co., Soesterberg, Netherlands).

### Cementation of the specimens

The specimens of the study groups were abraded at an air pressure of 0.2 MPa with 50 μm aluminum oxide particles (Aluminium oxide Eisenbacher Dentalwaren; ED GmbH, Woerth, Germany) for 10 s. The crowns were cemented on their corresponding abutments using transparent temporary cement (TempSpan™; Pentron Clinical, Wallingford, USA). The specimens were loaded under static load of 20 N during cementation [30]. All specimens were incubated in a water bath at 37 °C for 24 h before testing.

### Cyclic loading and thermocycling of the specimens

The crowns were held to a cyclic loading apparatus that was specially designed for this purpose. They were subjected to an average functional masticatory force of 10 KG, with a mean of 30,000 cycles. Thermomechanical aging was done using a custom-fabricated device for a total of 500 cycles representing 3 months of clinical service, between 5 and 55 degrees Celsius in water baths with dwell time of 1 min in each bath, and between the 2 baths, relaxation periods of 30s in air [31].

### Marginal fit evaluation

**Fig. 3:**
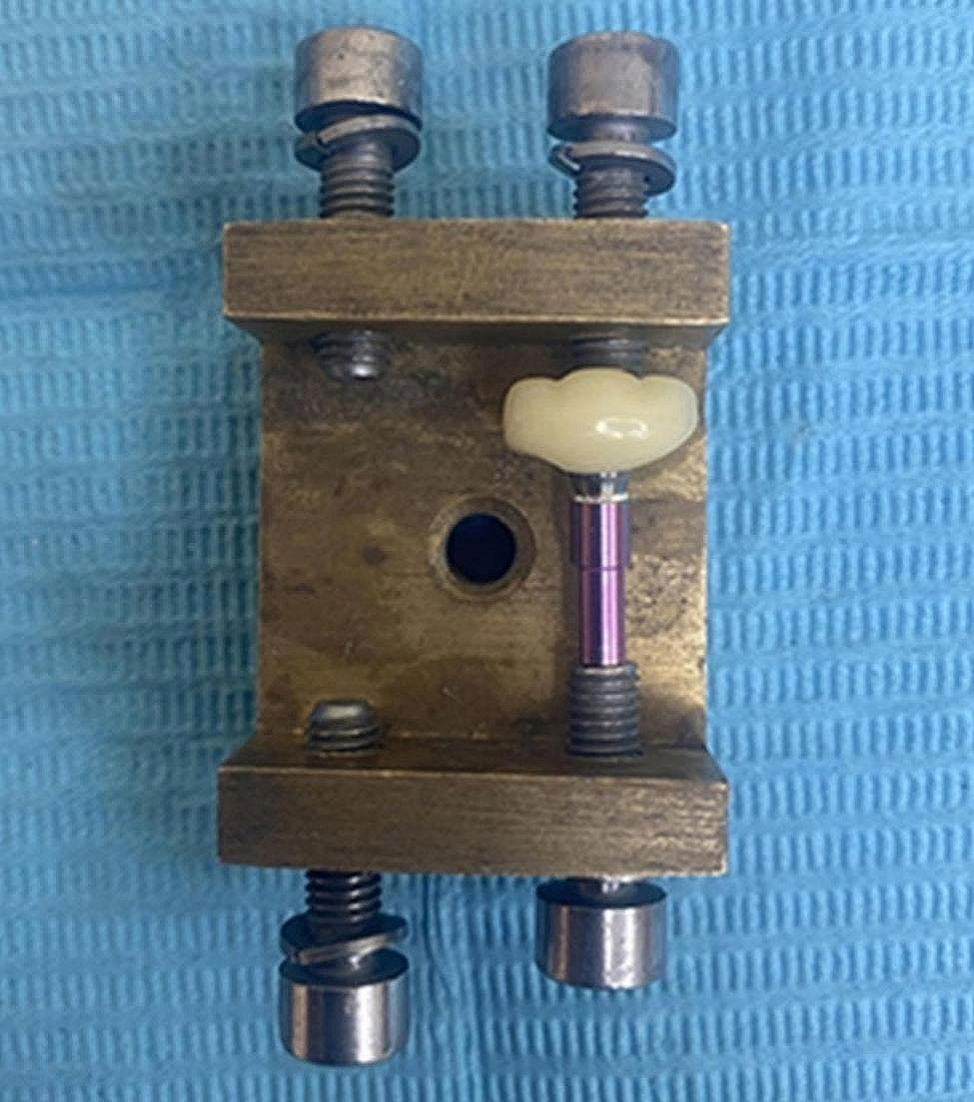
Holding device for marginal fit evaluation

**Fig. 4:**
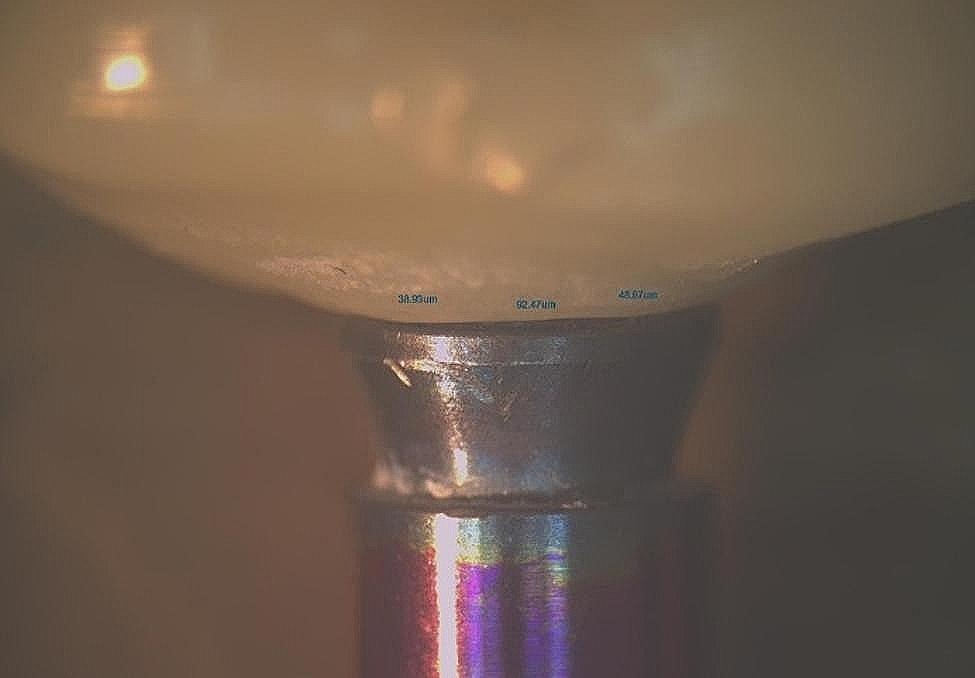
Marginal fit evaluation under stereomicroscope at 25× magnification

Marginal fit was assessed according to Holmes et al. evaluation for marginal gap [32]. Measurements were taken at a magnification of 25× using a stereomicroscope (Olympus SZ-1145TR Stereo ZoomMicroscope; OLYMPUS Co, Tokyo, Japan) connected to a digital camera. The crown and analogue were removed from the model and were held in place using a specially designed holding device (Fig. 3). Digital images were captured and processed with image analysis software (Toupview 3.7; Touptek Photonics, Zhejiang, China). Six measurements were obtained for each crown at mesiobuccal, midbuccal, distobuccal, mesiolingual, midlingual and distolingual points. A total of 36 measurements were gained from each group (Fig. 4). All measurements were carried out by a single blind operator with 10 years of experience with stereomicroscope. The crowns and digital analogues were fitted back into their corresponding 3D-printed master models.

### Fracture resistance test

**Fig. 5:**
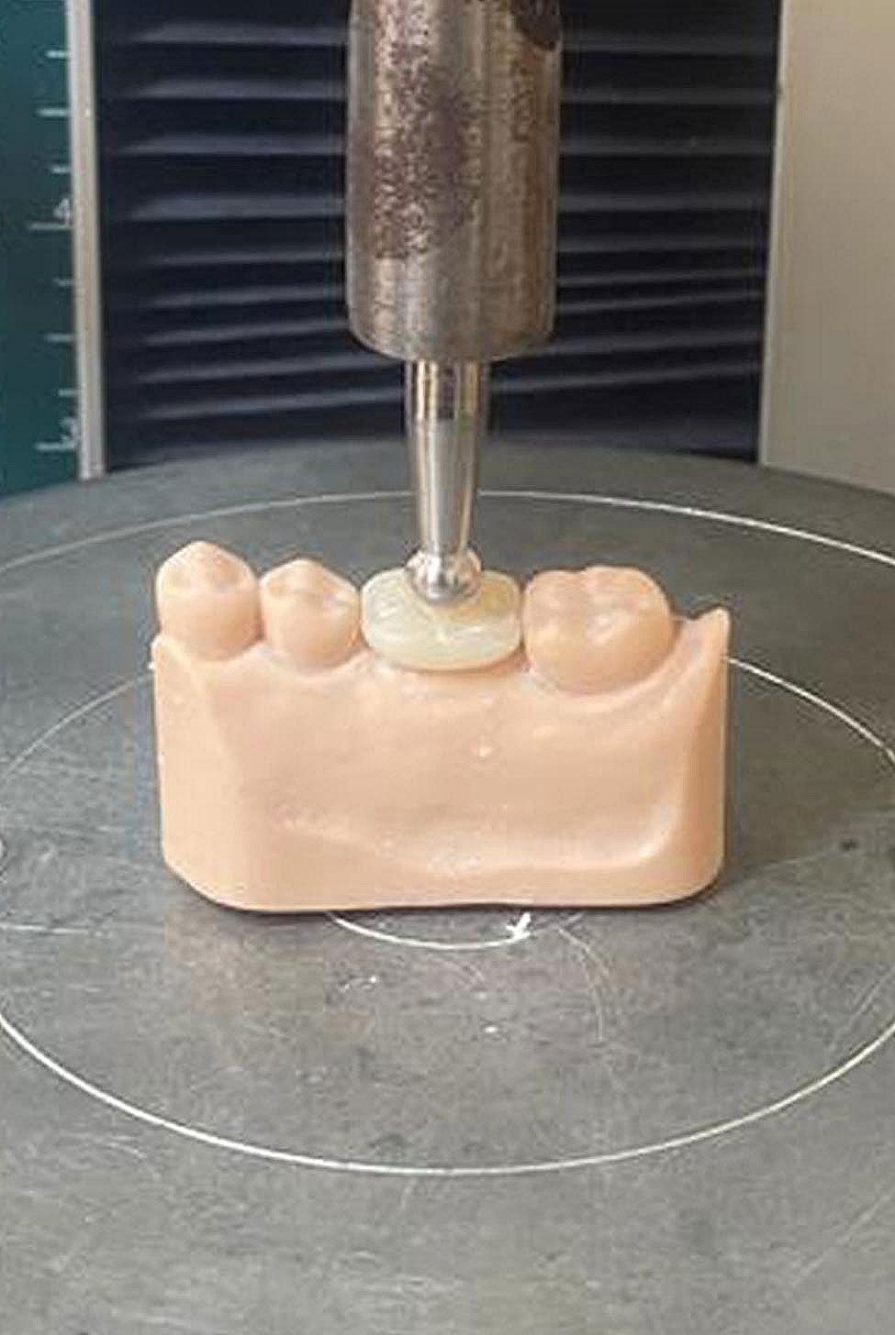
Specimen tested with universal testing machine for fracture resistance

The specimens were fitted back into the analogue spaces of the master models. The fracture resistance analysis was done by a universal testing machine (5ST; Tinius Olsen, UK). A custom-made stainless steel ball stylus with a diameter of 6 mm was used to apply the load perpendicularly on the implant axis at the central fossa at a crosshead speed of 1 mm/min until fracture (Fig. 5) [2]. Maximum loads were automatically recorded for each specimen in newtons (N) by software program (version 10.2.4.0; Horizon).

### Statistical analysis

Data were fed to a software package (IBM SPSS Statistics, v24.0; IBM Corp). The Shapiro–Wilk and the Kolmogorov-Smirnov tests were used to test the data normality. They revealed that the data of Marginal gap was non-parametric. To detect the significance between the study groups the Kruskal-Wallis test was used. The Dunn test with Bonferroni corrections was used for post hoc comparison of the groups (α = 0.05). The data of Fracture resistance test was parametric. To detect the significance between the study groups F-test (ANOVA) was used. The Tukey test was used for post hoc comparison of the groups (α = 0.05).

## Results

For marginal gap, Table 1 summarizes the descriptive statistical analysis of study groups. The Kruskal–Wallis test showed a significant difference between the study groups (*P* < .001). The Post hoc Dunn test showed that SM group and AM group had significantly lower marginal gap values compared to group CR (*P* = .003). There was no significant difference between SM group and AM group (*P* = .994).

**Table 1:** Measured gap values for study groups in µm

Group	Minimum	Maximum	Median ± SD	*P*	*P*_1_	*P*_2_
Group CR	41	204	83 ± 47.89	0.001*		
Group SM	8	60	18 ± 16.88		0.003*	
Group AM	10	44	15 ± 13.27		0.003*	0.994

* Indicates a significant difference; *P* indicates significance level between study groups according to Kruskal-Wallis test; *P*1 is value for comparison between group CR and group SM, and between group CR and group AM; *P*2 is value for comparison between group SM and group AM according to Dunn test

The highest mean marginal gap was detected in CR group with a median gap value of 104 ± 48 μm followed by AM group with a median gap value of 23 ± 13 μm and the SM group with a median gap value of (23 ± 17 μm).

For fracture resistance, Table 2 summarizes the descriptive statistical analysis of study groups. One-way ANOVA revealed a significant difference in fracture resistance between the study groups (*P* < .001). The Tukey Post hoc test showed that SM group had significantly higher fracture strength followed by AM group and CR group (*P* = .001).

**Table 2:** Measured fracture resistance values for study groups in N

Group	Minimum	Maximum	Mean ± SD	*P*	*P*_1_	*P*_2_
Group CR	706.71	801 − 60	739.52 ± 40.71	˂0.001*		
Group SM	1067.61	1202.65	1129.28 ± 57.86		0.001*	
Group AM	802.52	990.73	901.84 ± 72.11		0.001*	0.001*

* Indicates a significant difference; *P* indicates significance level according to ANOVA; *P*1 is value for comparison between group CR and group SM, and between group CR and group AM; *P*2 is value for comparison between group SM and group AM according to Tukey test

The highest mean ± SD fracture load value was detected in SM group (1129.28 ± 57.86 N), followed by AM group with a mean fracture load of 901.84 ± 72.11 N. The CR group had the lowest mean fracture load of 739.52 ± 40.71 N.

## Discussion

This study aimed to compare the marginal fit and fracture resistance of conventionally fabricated, subtractively milled, and additively 3D-printed implant-supported interim crowns. Both null hypotheses were rejected, as the fabrication techniques used significantly affected fracture resistance and marginal fit of study groups.

All tested groups showed clinically accepted marginal gap values according to McLean and von Fraunhofer [33]. However, conventionally fabricated crowns had significantly higher marginal gaps compared to milled and 3-D printed crowns. The autopolymerizing acrylic resin for the conventional interim crowns was reported to produce a high volumetric shrinkage (6%) which may affect the marginal fit [9]. In addition, manipulation of resin, trimming off the excess material and finishing can lead to distortion and misfit in the conventional interim crowns [10]. On the other side, pre-polymerized blanks for milling are made under high pressure and temperature. If there was shrinkage due to polymerization of the blank, it was reported to happen during its processing prior to milling [11]. In the 3D-printing, DLP technology was used, which is a highly precise process, where the resin polymerization process is performed in a cross-sectional manner [6]. Each layer is polymerized individually in the controlled conditions of the 3D printer, resulting in accurate reproduction of details, and minimizing volumetric shrinkage [12].

Direct evaluation of the marginal fit with a stereomicroscope was used to measure the vertical marginal gap as a non-invasive method, eliminating the need for specimen destruction and minimizing cumulative errors [23]. The use of digital analogues to fabricate interim crowns allowed direct measurement of marginal discrepancy. The cemented crowns on the corresponding abutments and laboratory analogues were easily detached from the printed models for measurement.

Fracture resistance and durability are critical elements in fabricating implant-supported interim restorations [31]. In the present study, all specimens of the three groups survived cyclic loading and thermocycling.

The milled crowns had significantly better fracture resistance followed by the 3D-printed and conventional interim crowns. The conventional fabrication of interim crowns is operator-dependent and subjected to manipulation errors affecting the mechanical properties of resultant crowns [7, 8]. The superior fracture resistance of milled interim crowns might be attributed to the optimum polymerization process of resin blanks under high pressure and temperature that leads to low residual monomer content, and long polymer chains [5, 9]. The resin-based standardized blanks are cross-linked into a three-dimensional structure that results in a more durable, less porous and stronger polymer [10].

On the other hand, printed crowns are liable to inconsistency and errors during polymerization, printing, and post-processing procedures [12].

Regarding fracture strength, the findings of this study agree with Martin-Ortega at al [4] who reported significantly higher fracture strength for milled implant supported interim crowns (423.8 ± 68 N) compared with printed crowns (423.8 ± 68 N). However, the values for fracture resistance reported in the study by Martin-Ortega et al. [4] were lower than the values of the current research, this might be attributed to the difference in materials brands and fabrication parameters applied in the two studies. For marginal and internal fit, the findings of the current research disagree with Park et al. [6] who reported significantly better marginal and internal fit for printed interim crowns compared with both milled and conventionally fabricated crowns. This difference might be attributed to different study set up in this research, as in the study by Park et al. [6], the specimens were assessed without thermomechanical aging using the replica method. Similarly, the results of the current study contradict the results of the study by Mohajeri et al. [29] who reported significantly better marginal fit for conventional crowns compared with both milled and printed crowns. Moreover, Mohajeri et al. [29] reported higher marginal gap mean values for milled and printed crowns (75.28 and 91.40 respectively). These differences might be a result of the differences in the methodology used in this study.

The study limitations included the in vitro design, the limited manufacturing procedures, and the materials tested. Future clinical studies with larger sample sizes, diverse materials, and long-term clinical data are recommended to build on these findings.

## Conclusions

Based on the findings of the current research, the following was concluded:

- Marginal adaptation and fracture resistance of implant-supported interim crowns are significantly affected by the fabrication method employed.
- Implant-supported interim crowns fabricated digitally, either through additive or subtractive methods, exhibited superior marginal adaptation compared to conventional method.
- The milled implant-supported interim crowns had better fracture resistance compared to 3D-printed and conventional crowns.

## Data Availability

The datasets generated and analyzed during the current study are available from the corresponding author on reasonable request.

## Abbreviations

mm: Millimeter
nm: Nanometers
°C: Celsius or centigrade
CAD/CAM: Computer-aided design & computer-aided manufacturing
no.: Number
mm/min: Millimeter per minute
IBM SPSS: Software for advanced statistical analysis
P: Probability
N: Newton
MPa: Megapascal
SD: Standard deviation
